# A9 INTESTINAL ORGANOID TRANSPLANTATION REVERSED THE INTESTINAL EPITHELIUM DAMAGE IN EXPERIMENTAL NECROTIZING ENTEROCOLITIS

**DOI:** 10.1093/jcag/gwac036.009

**Published:** 2023-03-07

**Authors:** B Li, C Lee, M Cadete, D Lee, H Zhu, P Sherman, A Pierro

**Affiliations:** The Hospital for Sick Children, Toronto, Canada

## Abstract

NOT PUBLISHED AT AUTHOR’S REQUEST

**Please acknowledge all funding agencies by checking the applicable boxes below:**

CIHR

**Disclosure of Interest:**

None Declared

